# Biological Functions and Therapeutic Potential of Lipocalin 2 in Cancer

**DOI:** 10.3390/ijms21124365

**Published:** 2020-06-19

**Authors:** Ginette S. Santiago-Sánchez, Valentina Pita-Grisanti, Blanca Quiñones-Díaz, Kristyn Gumpper, Zobeida Cruz-Monserrate, Pablo E. Vivas-Mejía

**Affiliations:** 1 Department of Biochemistry, University of Puerto Rico, Medical Sciences Campus, San Juan 00936, Puerto Rico; ginette.santiago@upr.edu (G.S.S.-S.); blanca.quinones@upr.edu (B.Q.-D.); 2 Department of Internal Medicine, Division of Gastroenterology, Hepatology, and Nutrition, The Ohio State University Wexner Medical Center, Columbus, OH 43210, USA; Valentina.PitaGrisanti@osumc.edu (V.P.-G.); Kristyn.Gumpper@osumc.edu (K.G.); 3 The James Comprehensive Cancer Center, The Ohio State University Wexner Medical Center, Columbus, OH 43210, USA; 4 University of Puerto Rico Comprehensive Cancer Center, San Juan 00935, Puerto Rico

**Keywords:** lipocalin 2, NGAL, siderophore, LCN2-MMP-9, cancer, oncogene

## Abstract

Lipocalin-2 (LCN2) is a secreted glycoprotein linked to several physiological roles, including transporting hydrophobic ligands across cell membranes, modulating immune responses, maintaining iron homeostasis, and promoting epithelial cell differentiation. Although LNC2 is expressed at low levels in most human tissues, it is abundant in aggressive subtypes of cancer, including breast, pancreas, thyroid, ovarian, colon, and bile duct cancers. High levels of LCN2 have been associated with increased cell proliferation, angiogenesis, cell invasion, and metastasis. Moreover, LCN2 modulates the degradation, allosteric events, and enzymatic activity of matrix metalloprotease-9, a metalloprotease that promotes tumor cell invasion and metastasis. Hence, LCN2 has emerged as a potential therapeutic target against many cancer types. This review summarizes the most relevant findings regarding the expression, biological roles, and regulation of LCN2, as well as the proteins LCN2 interacts with in cancer. We also discuss the approaches to targeting LCN2 for cancer treatment that are currently under investigation, including the use of interference RNAs, antibodies, and gene editing.

## 1. Introduction

Lipocalin-2 (LCN2), also known as neutrophil gelatinase-associated lipocalin (NGAL), siderocalin, uterocalin, and oncogene 24p3, is a secreted glycoprotein of the adipokine superfamily [1]. LCN2 exists as a ~25kDa monomer, a disulfide-linked homodimer, and a disulfide-linked heterodimer with matrix metalloproteinase 9 (MMP-9, gelatinase-B) [2,3,4,5]. LCN2 was first isolated from human neutrophils and described as an α_2_-microalbumin-related protein due to its homology with α_2_-microalbumin proteins from rats [2,3]. LCN2’s tertiary structure, determined by Nuclear Magnetic Resonance (NMR) spectroscopy, contains an N-terminal 3_10_-like helix, followed by eight antiparallel β-sheets enclosed by two α-helices that form a cup-shaped pocket [6].

LCN2’s function was first identified as an acute-phase protein secreted from neutrophils during bacterial infections [7]. In this regard, Goetz et al. noted that LCN2 isolated from human neutrophils bound to the iron-loaded bacterial siderophore enterochelin [7]. Enterochelin and other siderophores are secreted by bacteria to ensure iron supply for their cellular processes [8]. Adding LCN2 to a bacterial culture impedes bacterial growth through its iron/siderophore-binding capabilities [7]. Further studies have found that many bacterial siderophores from Gram-negative bacteria, Gram-positive bacteria, and mycobacteria also bind to LCN2 [9,10]. NMR spectroscopy identified the ligand-binding region for siderophores and hydrophobic molecules in a region called the lipocalin fold (amino acids 48–136), located in LCN2’s cup-shaped pocket [11,12,13].

LCN2 can also interact with mammal-derived siderophores [14,15]. By interacting with mammalian siderophores, LCN2 acts as an iron carrier and maintains the iron levels intracellularly and extracellularly. For example, Bao et al. showed that LCN2 delivers catechol-bound iron from the extracellular space to the cytoplasm [10,12]. Once in the cytoplasm, iron can mediate expression of iron-responsive genes, including ferritin and transferrin receptor [16].

Dysregulation of LCN2 has been tied to obesity, metabolic syndrome, and cardiovascular diseases, mainly through its ability to bind to lipids like fatty acids [17]. For example, LCN2 can bind to the fatty acid retinoic acid to mediate thermogenesis and lipid metabolism in adipose tissue [18]. Additionally, downregulation of LCN2 in mice attenuates the metabolism of arachidonic acid, impairing energy homeostasis [19]. Because obesity is a risk factor for many cancers, LCN2 has become a focus of cancer research.

Humans express LCN2 in multiple tissues, such as adipose [18], lymphatic (bone marrow and spleen) [20,21], respiratory (bronchus, nasopharynx), digestive (stomach and salivary gland) [22], genitourinary (cervix and uterus), endocrine [23], muscle [24], and other immune cells in addition to the previously identified neutrophils [25]. Several studies have revealed that neoplastic tissues originating from the pancreas, colon, breast, and lung express elevated LCN2 levels when compared to normal tissues [25]. This finding has led to studies associating LCN2 overexpression with the progression of multiple cancers and with the poor prognosis of aggressive forms of breast cancer [26], pancreatic cancer [27], and endometrial carcinoma [28]. Because current treatment options for aggressive cancers are limited, LCN2 may be a promising therapeutic target against these cancer subtypes.

In this review, we summarize the most relevant findings regarding the expression of LCN2 in neoplastic tissues of several cancers and the molecular mechanisms leading to LCN2 overexpression in cancerous cells. We also discuss the biological and molecular consequences of LCN2 dysregulation in many tumor types, and we explain how its interaction with MMP-9 promotes cancer cell growth and metastasis. Finally, we describe the approaches to targeting LCN2 for cancer treatment that are currently under investigation, such as gene editing, interference RNA, and antibody-based therapy.

## 2. LCN2 Expression in Cancer

LCN2 expression levels are particularly high in breast, pancreas, ovarian, colorectal, thyroid, and bile duct cancer tissues and cell lines derived from these tumors [27,29,30,31,32,33,34,35]. Table 1 summarizes various studies reporting LCN2 expression levels and LCN2′s function in several cancer types.

Stoesz et al.’s study was the first to report high expression levels of LCN2 in primary breast cancers [31]. In this study, the LCN2 protein levels were quantified in 28 breast tumors by immunohistochemistry (IHC). The breast tumors were low to high grade, with different histologic subtypes and steroid receptors [31]. Although the study did not include normal breast epithelia samples for comparison purposes, 39% of the samples showed a cytoplasmic positive LCN2 staining. In this same study, 109 (44%) of the 250 breast tumors analyzed via Western blot also exhibited the presence of LCN2 [31]. Other studies have validated these results by confirming LCN2’s presence in well-characterized invasive breast cancer tissues, urine of metastatic breast cancer patients, and breast cancer cell lines [26,36]. Interestingly, in breast cancer, increased LCN2 protein levels are associated with decreased disease-free survival, decreased disease-specific survival, and decreased overall survival [35,36].

In a gene expression profile study performed with 19 pancreatic cancer cells and the immortalized human pancreatic ductal epithelial cell line (HPDE), LCN2 was one of the genes significantly overexpressed in cancerous versus immortalized cells [37]. Similarly, LCN2 expression was found to be highly upregulated in human pancreatic cancer tissues and blood compared to pancreas tissue and blood samples of healthy controls [27,38]. In colon cancer, Nielsen and coworkers performed IHC analysis of 14 colorectal adenocarcinomas containing both malignant and normal epithelium. All malignant samples stained strongly positive for LCN2, compared to the epithelial cells from normal surrounding tissues, which were negative for LCN2. In situ hybridization revealed a strong signal for LCN2-mRNAs in 10 of the 11 colon cancer samples tested. In addition, 30 colorectal tissues derived from inflammatory disorders (appendix, diverticulitis, ulcerative colitis, and Crohn’s disease) were analyzed by in situ hybridization, showing a strong signal for the expression of LCN2-mRNAs in 20% of the samples. However, there were no significant differences in the expression of LCN2 between neoplastic and non-neoplastic tissues [29]. In the thyroid, IHC analysis of different thyroid tissues showed that tissues derived from papillary, follicular, and anaplastic thyroid carcinomas had significantly higher levels of LCN2 expression compared to normal thyroid tissue [33]. In fact, the study showed that increased LCN2 levels were proportional to the malignant phenotype of these tumors, the anaplastic thyroid carcinoma being the one with the highest LCN2 expression [33]. The increased expression of LCN2 in colon and thyroid tumor tissues has also been confirmed using public microarray datasets [29,33]. Furthermore, increased expression of LCN2 has been reported in the tumor tissues and cell lines of the highly aggressive cholangiocarcinoma [34].

**Figure 1:**
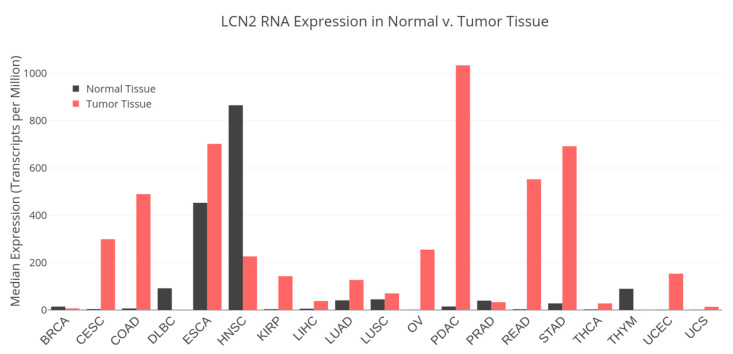
LCN2 differential RNA expression in normal and tumor tissue. The Cancer Genome Atlas (TCGA) RNA-Seq datasets from healthy and tumor tissue. BRCA: breast invasive carcinoma; CESC: cervical squamous cell carcinoma and endocervical carcinoma; COAD: colon adenocarcinoma; DLBC: lymphoid neoplasm diffuse large B-cell lymphoma; ESCA: esophageal carcinoma; HNSC: head and neck squamous cell carcinoma; KIRP: kidney renal papillary cell carcinoma; LIHC: liver hepatocellular carcinoma; LUAD: lung adenocarcinoma; LUSC: lung squamous cell carcinoma; OV: ovarian serous cystadenocarcinoma; PDAC: pancreatic ductal adenocarcinoma; PRAD: prostate adenocarcinoma; READ: rectum adenocarcinoma; STAD: stomach adenocarcinoma; THCA: thyroid carcinoma; THYM: thymic carcinoma; UCEC: uterine corpus endometrial carcinoma; UCS: uterine carcinoma.

An interrogation of The Cancer Genome Atlas (TCGA) data portal showed that several cancers have significantly altered expression of LCN2 compared to normal tissue, suggesting LCN2’s potential as a prognostic biomarker [39] (Figure 1). Some studies have correlated upregulated LCN2 expression in tumor tissue with poor outcomes caused by increased growth of cancer cells, therapeutic resistance, invasion, and metastasis [27,28,36,40,41]. Similarly, Bauer et al. analyzed 207 well-characterized breast cancer tumor tissues by IHC and observed a significant correlation between high LCN2 levels and negative estrogen receptor expression [36]. Moreover, high LCN2 levels showed a significant correlation with HER-2 overexpression and a high Ki-67 proliferation index [36]. These results suggest a strong association between high levels of LCN2 and poor-prognosis-associated markers in breast tumors. Furthermore, high expression of LCN2 or the LCN2-MMP-9 heterodimer is associated with worse outcomes in patients with gastric, breast, and high-grade endometrial cancers [35,36,42,43]. The use of LCN2 as a prognostic marker seems to be promising; however, more studies with larger samples of cancer patients and normal controls are required. It is also important to fully understand the role of LCN2 in cancer progression (see Section 3) before proposing LCN2 as a truly diagnostic and/or prognostic marker for certain cancer types (i.e., aggressive cancers).

## 3. Functions of LCN2 in Cancer

In the last decade, the biological role of LCN2 in carcinogenesis has been addressed using mostly cancer cell lines and mouse models. The majority of these studies agree that LCN2 upregulation promotes tumor progression and metastasis. For example, in breast cancer, increased LCN2 levels promoted cell proliferation and angiogenesis [43,56]. Other studies have shown that high levels of LCN2 correlate with epithelial-to-mesenchymal transition (EMT), invasion [33,34,36], and metastasis [26] in different tumor types. Additionally, LCN2 overexpression and its iron-shuttling functions have been associated with the inhibition of apoptosis, which is essential for cancer cell survival in the tumor microenvironment (TME) [13,57]. In this section, we discuss the molecular and biological effects of LCN2 dysregulation in cancer cells, and we explain how the association of LCN2 with iron and the LCN2–MMP-9 interaction contributes to cancer progression. Findings that high levels of LCN2 are associated with inhibition of tumor growth and proliferation are also discussed.

### 3.1. LCN2 as a Promoter of Cancer Progression

Elevated LCN2 expression is strongly associated with the acquisition of a metastatic phenotype of cancer cells [58]. Overexpression of LCN2 in MCF-7 breast cancer cells, which normally express low LCN2 levels and exhibit an epithelial phenotype, induced the transition to a mesenchyme-type morphology. LCN2 overexpression also increased the expression of classic mesenchymal markers, such as vimentin and fibronectin, and increased cell migration and invasion [26]. Orthotopic implantation of LCN2-overexpressed MCF-7 breast cancer cells in mice increased local tumor invasion and growth when compared to parental MCF-7 cells [26]. These findings are in agreement with studies by Shi and coworkers, who overexpressed LCN2 in the aggressive breast cancer cell line 4T1 and observed increases in migration and invasion of these cells compared to 4T1 parental cells [59]. LCN2-siRNA-based silencing in the highly aggressive and invasive breast cancer cell line MDA-MB-231 decreased cell migration and suppressed the mesenchymal phenotype [26]. In thyroid cancer, colony formation assays revealed a decrease in cell proliferation following LCN2-siRNA-mediated silencing in LCN2-overexpressing human anaplastic thyroid cancer cells (FRO cells) [33].

Additional evidence from mouse models supports the notion that LCN2 promotes cancer cell progression and metastasis. Studies of transgenic breast cancer mouse models that have a biallelic Lcn2 deficiency have shown a delay in tumor-formation rates, metastasis, and the number and size of primary tumors compared to normal or Lcn2 heterozygous mice [60,61]. A subcutaneous mouse model in pancreatic cancer showed that upregulation of LCN2 promotes invasion, tumorigenicity, and gemcitabine resistance [51]. Moreover, using an Lcn2 whole-body knockout mouse model, it was shown that lack of Lcn2 expression reduced extracellular matrix deposition, extended survival, and delayed tumor growth in both an obesity-driven genetic and an orthotopic mouse model of pancreatic cancer [27]. In a thyroid cancer mouse model, mice injected with LCN2-silenced FRO cells had lower tumor volumes, had lower tumor weights, and developed fewer tumors overall than the mice injected with nontransfected FRO cells [33].

Interestingly, in human breast cancer, LCN2 levels have been found to be elevated at advanced cancer stages. For example, there are significantly higher levels of LCN2 in breast tumor tissues (stages II–III) compared to normal breast stroma [26]. Because breast cancer stages II–III are characterized by extravasation to lymph nodes and distant organs, high levels of LCN2 seem to promote the invasive potential of breast cancer cells [26]. Moreover, LCN2 expression is increased in neoplastic pancreatic tissue lesions, which typically develop into pancreatic cancer [38].

### 3.2. LCN2 Association with Iron Trafficking and Tumor Progression

**Figure 2:**
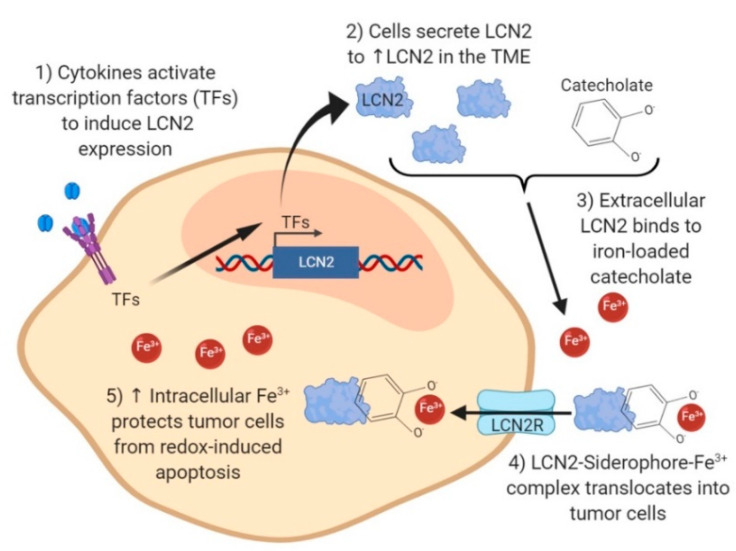
Intracellular iron regulation by LCN2 in tumor cells. Cytokines (IL-17, IL-1β, and TNF-α) secreted by immune cells in the tumor microenvironment (TME) induce activation and binding of transcription factors (TFs) NF-κB, AP-1, PU.1, GATA-1, or C/EBPβ to the LCN2 promoter region. Transcription and secretion of LCN2 is stimulated. LCN2 in the cytoplasmic space binds to iron-loaded catecholate and re-enters tumor cells as an LCN2-catecholate-Fe^3+^ complex through the LCN2 receptor (LCN2R) expressed in tumor cells. Increased intracellular iron protects tumor cells from ROS-induced damage and from apoptosis, and it provides resistance to hypoxic conditions.

LCN2 plays a pivotal role in intracellular iron homeostasis through its interaction with siderophore-like molecules, such as catechol [10]. The interaction of LCN2 with catechol and iron may explain the advantaged survival of tumor cells in which LCN2 is upregulated [13,62,63]. Figure 2 summarizes some of the major pathways associated with the LCN2–catechol interactions in tumor cells. This interaction activates iron trafficking, the regulation of iron-responsive genes, and cancer progression [10,13,16]. Cytokines secreted by the TME, such as interleukin (IL)-17, IL-1β, and tumor necrosis factor (TNF)-α, stimulate the transcription of LCN2 by inducing the activation and binding of transcription factors such as NF-kB, AP-1, PU.1, GATA-1, or C/EBPβ to the LCN2 promoter region [64,65]. Once expressed and secreted, LCN2 binds to catecholate in the extracellular space to form the complex LCN2-catecholate [10]. Catecholate bound to LCN2 harvests iron (Fe^3+^) from the extracellular iron pool and enters the cell through LCN2 receptors (LCN2Rs) [13,66]. High levels of LCN2Rs allow the entry of LCN2-catecholate-iron molecules, increasing intracellular iron concentration [13].

The LCN2-catecolate-Fe^3+^ complex in the intracellular space has two major effects in tumor cells: a decrease in free catechol levels and an increase in iron (Fe^3+^) levels. The hydroxyl group in the free catechol undergoes O-hydroxylation or O-sulfonation, reducing Fe^3+^ to Fe^2+^ and increasing hydroxyl-radical formation [1]. Iron redox reaction and hydroxyl radical species are molecules essential to activating the Fenton reaction, which causes oxidation of molecules and further biological damage in the cell [67]. Therefore, the decrease in free catechol inside cells prevents the production of reactive oxygen species (ROS) and the survival of tumor cells [10].

High intracellular iron levels induced by the LCN2-catecholate-Fe^3+^ complex suppress apoptosis of tumor cells by blocking the induction of the pro-apoptotic protein Bim [68]. Normally, Bim activates apoptosis via Bax/Bax-mediated cytochrome c release [68]. This idea is supported by a study conducted by Roudkenar et al., who observed apoptosis inhibition in clones with high levels of LCN2 after treatment with H_2_O_2_ [63]. The inhibition of apoptosis by the increase in iron levels is also promoted by a decrease in iron efflux. The increase of iron (Fe^3+^) ions in the intracellular space of tumor cells leads to a switch in the ferroportin–hepcidin regulatory axis on the basis of an unknown mechanism [69]. Ferroportin is the only known iron ferrous exporter [70], and high levels of intracellular iron trigger ferroportin lysosomal degradation, resulting in a decrease of iron efflux [69]. Moreover, increased levels of iron in cancer cells trigger stabilization of the hypoxia-inducible factor 1 alpha (HIF-1), promoting changes in glycolysis, cell division, and cancer cell stemness, leading to cancer cell survival and metastasis [71,72]. In general, the proposed mechanism suggests that the ternary complex (LCN2-catecholate-Fe^3+^) promotes tumor cell growth, prevents tumor cell apoptosis, provides resistance to hypoxic conditions, and protects tumor cells from an environment of oxidative stress [10,13,71]. Because LCN2’s binding to catechols and iron transport may be essential to tumor cell survival, blocking these interactions is a potential therapeutic target for inhibiting cancer progression.

Moreover, because iron dependency is a hallmark of cancer cells, the association of LCN2 upregulation with the ferroptosis cell-death mechanism must be addressed. Ferroptosis, an iron-catalyzed form of regulated necrosis, occurs due to excessive peroxidation of polyunsaturated fatty acids [73]. Thus, intracellular iron accumulation caused by the LCN2-cathecolate-Fe3+ ternary complex may also increase iron-dependent lipid-derived ROS accumulation through the Fenton reaction, accelerating ferroptosis. However, the antioxidant glutathione peroxidase 4 (GPX4) and the ferroptosis suppressor protein 1 (FSP1) suppress the production of lipid-derived ROS. Therefore, the secretion of GPX4 and FSP1 antagonizes the ferroptosis process and may enable cancer cells to bypass ferroptosis. This hypothesis is supported by evidence showing the production of antioxidant enzymes such as heme oxygenase (HO-1) and superoxide dismutases (SO-1,2) in LCN2-overexpressing cancer cells [74]. Thus, it is crucial to investigate ferroptosis by manipulating the LCN2 expression levels in cancer cells before proposing this molecular pathway as a target for therapy.

### 3.3. LCN2–MMP-9 Interaction Promotes Cancer Cell Invasion and Metastasis

Several studies have observed high levels of LCN2 associated with MMP-9 as a heterodimer in the urine of breast cancer patients, in high-grade endometrial cancer tissues, and in cell lysates of oral squamous carcinoma cell lines [35,43,75]. The interaction of LCN2 with MMP-9 seems to play a crucial role in the modulation of the metastatic phenotype of cancer cells. High levels of monomeric forms of LCN2, MMP-9, and LCN2-MMP-9 heterodimers are secreted into the extracellular space, and their levels seem to correlate with the aggressive behavior of neoplastic cells in several types of cancer [42,43,75,76,77]. The complex prevents MMP-9 degradation in a dose-dependent manner, a mechanism that protects the MMP-9 catalytic activity [77]. Data support the use of high-molecular-weight matrix metalloproteinases (MMPs), such as the LCN2-MMP-9 complex, as independent predictors of metastatic phenotypes in several cancers, including prostate and bladder cancers [78].

Pro-inflammatory cytokines, such as IL-17 and TNF-a, and proteins such as C-X-C chemokine receptor type 7 (CXCR7) activate the secretion of MMP-9 or LCN2 in the TME, contributing to the metastatic phenotype. For example, in breast cancer, upregulation of CXCR7 regulates metastasis by enhancing the expression of the adhesion molecule vascular cell-adhesion molecule (VCAM)-1 and MMPs such as MMP-2 and MMP-9 [79]. Moreover, cytokines, such as IL-17 and TNF-a, activate transcription of LCN2, and stroma-derived LCN2 enhances the malignant phenotype of breast cancer cells and promotes cell metastasis, possibly by interacting with MMP-9 [80]. Additionally, immune cells and fibroblasts from the tumor stroma produce a variety of inflammatory cytokines, ROS, chemokines, and proteases, which affect cancer progression [81,82].

In cancer cells that exhibit LCN2 overexpression, LCN2’s modulation of MMP-9 seems to be crucial for the invasion of malignant cells through the basement membrane; this modulation occurs through three different mechanisms. First, LCN2 protects pro-MMP-9’ s degradation of its natural inhibitor, tissue inhibitor of metalloproteinases 1 (TIMP-1), as seen by a reduction in MMP-9’s degradation after incubation with LCN2 in a dose-dependent manner [77]. In this study, MMP-9 and TIMP-1 transcript levels did not decrease after LCN2 incubation, suggesting that MMP-9’s activity is modulated by LCN2, not by a decrease in the enzyme levels. However, further research must be conducted to determine whether LCN2 modulates the activity of MMP-9 in vivo. Second, LCN2 allosterically regulates pro-MMP-9 by inducing pro-MMP-9 activation, similarly to other MMP-9 inducers, such as HgCl_2_ and plasma kallikrein [83]. The proposed mechanism suggests that LCN2 activates pro-MMP-9 allosterically by trapping the N-terminal of the pro-MMP-9 inside LCN2’s binding pocket, resulting in the opening of the active site of pro-MMP-9 and full activation of the enzyme [83]. Lastly, the third mechanism suggests that LCN2 increases the enzymatic activity of MMP-9 [34]. A study of cholangiocarcinoma cells observed a decrease in MMP-9 activity, but not in its mRNA levels, after inducing the silencing of LCN2 [34].

### 3.4. High LCN2 Levels Correlate with Reduced Tumor Growth in Certain Cancer Types

Some studies have suggested that LNC2 acts as a tumor suppressor [38,44,46,79]. For example, a study in hepatocellular carcinoma (HCC), a very aggressive primary liver cancer, performed ectopic expression of LCN2 in HCC cell lines (Chang liver and SK-Hep-1) and observed a significant inhibition in cell proliferation, migration, and invasion [79]. However, the same study performed gene expression analysis using publicly available databases like GEO1898, GSE4024, and Oncomine and found a significantly higher expression of LCN2 mRNA in HCC tumors than in nontumor tissue [79]. Similarly, Tong et al. reported decreased cell adhesion and invasion after LCN2 overexpression in various pancreatic cancer cells [50]. However, a tissue expression microarray demonstrated intense luminal and cytosolic staining of LCN2 in human pancreatic cancer tissues, whereas normal pancreatic ductal epithelial cells showed no LCN2 expression. A study of colon cancer showed that high levels of LCN2 in supernatants of primary and metastatic colon cancer cells inversely correlate with their metastatic potential [38]. An orthotopic xenograft mouse model of pancreatic cancer showed a decrease in tumor size, metastatic score, and the expression of the CD31 angiogenesis marker in the LCN2-overexpressing mice group compared to the control group (non-LCN2-expressing pancreatic cancer cells) [50]. Finally, a study of breast cancer showed that overexpression of LCN2 in 4T1-H-Ras-transformed cells (4T1-R) reverted the mesenchymal phenotype of the 4T1-R cells to a more epithelial phenotype by increasing E-cadherin, reducing vimentin, and suppressing cell invasion [56]. Concomitant with the reversal phenotype of these cells, a suppression of the Ras-MEK signaling pathway was observed. In addition, Ras-induced Raf-, MEK-, and ERK1/2-phosphorylation was abrogated once Lcn2 was overexpressed in 4T1-R cells [56].

The idea that high levels of LCN2 inhibit tumor growth is supported by the study conducted by Lee et al., who overexpressed LCN2 in HCC cells and observed an inactivation of the c-Jun N-terminal kinase (JNK) and phosphatidylinositol 3’-kinase (PI3K)/Akt signaling pathway [84]. Inactivation of these pathways was accompanied by a decrease in proliferation and invasion of HCC cells [84]. Likewise, pancreas cancer cells that overexpressed LCN2 exhibited a reduction in focal adhesion kinase (FAK) tyrosine-397 phosphorylation and vascular endothelial growth factor (VEGF) secretion [50]. The reduction in FAK phosphorylation and VEGF secretion caused a reduction in cell adhesion and cell invasion.

In summary, whereas the studies discussed in this section suggest that LCN2 may play a role as a tumor suppressor, most in vitro and in vivo studies in the literature support the role of LCN2 as a tumor promoter (Table 1). The LCN2 field of study would benefit from larger-sample-size studies that further evaluate the aberrant signaling pathways in LCN2-overexpressing cancer tissues. Moreover, the use of murine-derived cells, such as 4T1 cells, has some limitations, including the absence of an LCN2 binding region for MMP-9 binding [80]. Thus, in these cells, neither the LCN2–MMP-9 heterodimer formation nor the activation of the LCN2 downstream signaling pathways occurs. Experiments using patient-derived xenograft mouse models to clarify whether LNC2 is a driver or suppressor of cancer cell growth are also needed.

## 4. Targeting LCN2 for Cancer Therapy

The study of LCN2 as a therapeutic target in cancer is still in the early stages of development. Recently, there has been increasing interest in the development of LCN2-based therapies; however, developing small molecule inhibitors represents a challenge, because LCN2 is a nonenzymatic protein. Current strategies under investigation to target LCN2 include LCN2 gene manipulation through gene editing techniques, post-transcriptional regulation through RNA interference, and protein regulation using targeted antibodies. Moreover, targeting the molecular processes directly associated with LCN2 may indicate an approach to mitigating LCN2-induced cancer development.

### 4.1. Targeting LCN2 through Gene Editing

The CRISPR-Cas9 gene editing system has considerable potential for directed therapeutic strategies. The use of CRISPR/Cas9-based gene knockouts requires RNA-guided Cas9 nuclease to conduct specific DNA strand breaks on a specific DNA sequence. Effective carriers for delivering either the plasmids containing the guide RNA (gRNA) and Cas9 DNA sequences or the gRNA/Cas-9 ribonucleoprotein complex are needed. To knock out the LCN2 gene from triple-negative breast cancer cells, Guo et al. [85] developed a DOPC (1,2-Dioleoyl-sn-Glycero-3-Phosphocholine)-based nanolipogel formulation and alginate hydrogel to co-deliver plasmids with gRNAs and Cas9 sequences. Three different CRISPR plasmids were encapsulated in DOPC and sequestered within alginate hydrogel conjugated to an intercellular adhesion molecule (ICAM-1) antibody for breast cancer cell specificity [85,86]. The combination of alginate as a noncationic, low-toxicity component in the nanoformulation decreases the possible cationic charge-induced toxicity of the nanoparticles [85,87]. The delivery of this gene editing system allowed for a specific interaction between the nanoparticles and the triple-negative breast cancer cells, resulting in a knockout of the LCN2 gene. This treatment reduced cell migration in vitro and tumor growth in vivo. More in vivo tissue distribution and safety studies are needed to assess the potential off-target effects commonly associated with the CRISPR/Cas9 gene editing approach. Optimizing nanocarriers for the CRISPR/Cas9 delivery system could lead to the development of therapies with lower toxicities compared to the standard chemotherapeutic regimens. The identification of specific tumor receptors of each cancer type in which LCN2 is overexpressed could also improve the specificity of the CRISPR/Cas9-containing nanocarriers [85].

### 4.2. Targeting LCN2 with RNA Interference

Short-interfering RNAs (siRNAs) have become a popular and effective strategy for achieving precise knockdown (silencing) of genes at the post-transcriptional level. This technology is particularly useful for targeting proteins with a planar surface, which are often considered “undruggable” Designing small molecule inhibitors for these proteins involves extensive and cumbersome work. SiRNA-mediated silencing of LCN2 has been tested in breast cancer [26] and cholangiocarcinoma (CCA) cells [34]. Nuntagowat et al. transfected Lcn2-siRNAs into the RMCCA-1 CCA cells and observed a significant reduction in cell invasion and cell migration, but not in cell proliferation [34]. In another study, Guo et al. prepared a pH-dependent nanoliposomal formulation containing LCN2-siRNA and a C-X-C chemokine receptor type 4 (CXCR4) inhibitor [88]. CXCR4 is a cell membrane receptor commonly found on the surface of breast cancer cells. CXCR4 activates the PI3K and Rho GTPase intracellular pathways, both of which are widely associated with metastatic breast cancer. The dual formulation, when incubated with metastatic breast cancer cells, significantly reduced the migration of these cells compared to adding each drug individually [88]. This research group decorated LCN2-siRNA-containing liposomes with an ICAM-1 antibody [89]. Incubation of triple-negative breast cancer cells with this formulation led to lower levels of VEGF and decreased angiogenesis in vitro [89]. Administration of conditioned media from triple-negative breast cancer cells pretreated with the liposomal formulation reduced angiogenesis in a chick chorioallantoic membrane assay [89]. Even though these studies are promising, more pharmacokinetic, pharmacodynamic, and biosafety studies are needed before moving these therapies into clinical trials.

### 4.3. Inhibiting LCN2 at the Protein Level

Recently, monoclonal antibodies have increasingly been used as an important therapeutic strategy for many diseases, including cancer [90]. Their high specificity allows for the advantage of minimum off-target effects and reduced potential side effects. As a secreted protein, LCN2 is a viable target for the development of antibody-based therapies. To demonstrate the therapeutic potential of using antibodies against the secreted LCN2 on breast cancer metastasis, Leng et al. [60] systemically administered LCN2 monoclonal antibodies in mammary-tumor-bearing mice. Antibody inoculation once per week for four weeks significantly reduced lung metastases as compared to the control groups [60]. These results showed that it is possible to block long-distance metastasis using specific antibodies against the secreted form of LCN2. However, the therapeutic efficacy and safety of LCN2-antibody-based therapy will need to be evaluated in clinical trials, most likely in combination with current therapies for each cancer type being targeted.

### 4.4. Targeting LCN2-Related Pathways

Targeting the major LCN2-associated pathways, including iron transport and/or MMP-9 activation, has yet to be undertaken. However, iron-chelating strategies and MMP-9 small inhibitors for cancer therapy have been addressed independently. For instance, targeting the iron dependency of cancer cells using chelating agents to sequester iron has reached clinical trials for cancer treatment. In particular, the iron chelators triapine, ciclopirox, and deferasirox effectively reduced the white-blood-cell count of patients with hematological malignancies [91]. Alternatively, approaches to mitigating iron dependency using agents that rely on increased iron levels and induce cell death have been tested. For example, ascorbate and ferroptosis inducers (i.e., sorafenib, sulfasalazine, and artesunate), which require iron to promote cell death, have been used in multiple cancers [92]. Moreover, high levels of iron allow for the active targeting of cancer cells through the transferrin receptor, as explained above [91]. On the other hand, the activity of MMP-9 can be blocked by direct inhibition of its protease activity or by inhibiting its expression. Although chemical inhibition of MMP-9 using broad-spectrum inhibitors seemed promising for cancer treatment, most of the inhibitors failed in the clinic due to adverse effects. However, selective MMP-9 inhibitors, such as hydroxamates, pyrimidine-2,4,6-triones, carboxylic-acid-based inhibitors, and others, have been tested as potential cancer therapies [93]. Moreover, monoclonal antibodies against MMP-9 have had success, particularly in colorectal cancer [93,94]. It would be interesting to investigate how LCN2 is modulated when iron-transport and/or MMP-9 pathways are targeted. Combination therapies targeting LCN2, iron-level regulation, and MMP-9 activity could produce synergistic cancer cell death.

## 5. Concluding Remarks

LCN2 expression levels are clearly modulated in many types of cancers. LCN2 appears to promote tumorigenesis by increasing invasion, metastasis, and proliferation while decreasing apoptosis. Some of these characteristics are the result of LCN2′s ability to facilitate iron intake to cancer cells or its ability to form a heterodimer with MMP-9. However, there is also evidence suggesting that LCN2 could act as a tumor suppressor by inhibiting proliferation and invasion. Nonetheless, there are more data (from cell lines, mouse models, and humans) that identify LCN2 as a tumor-promoter molecule. The discrepancy in the results demonstrates the need for further studies to clarify the various factors that potentially modulate the function of LCN2 in cancer. For the same reason, the prognostic value of LCN2 may vary depending on confounding factors, but its increased level among numerous cancers makes LCN2 a promising biomarker for this disease.

Targeting LCN2 has been shown to be beneficial in different mouse cancer models. This research area is still under development, and further studies are necessary to determine its efficacy as well as the circumstances under which this type of therapy would be most advantageous. Understanding the role of LCN2 will be essential to determining its potential as a biomarker or as a cancer therapy target.

## Figures and Tables

**Table 1:** Expression and Function of LCN2 in Cancer.

Cancer	Sample Type	LCN2 Expression	Function/Clinical Correlation	Refs
Breast	Cell Lines: MCF-7, aggressive MDA-MB-231, T47D:A18, T46D:C4:2W	↑	Promotes EMT	[26,31]
	Tumor Tissue	↑	Poor Prognosis in ER^−^/PR^−^/HER2^+^ tumors, Lymph Node Metastasis	[31,36]
	Urine	↑	Possible Biomarker for Cancer	[26]
	Orthotopic Xenograft	↑	↑ Invasion, ↑ Lymph Node Metastasis	[26]
	Cancer Patients	↑	↓ DSS, ↓DFS	[36]
Cholangiocarcinoma	Cell Lines: RMCAA-1, RBE, SSP-25, TFK-1, SNU308, SNU1079, TGBC-24, HUCCT1, YSCCC	↑	↑ Metastasis, ↑ Proliferation	[34,40]
	Tumor Tissue	↑	Poor Prognosis	[34]
Colon	Cell Lines: KM12C, SW480, JCT116, JT29, LS174T, KM12SM, SW620, COLO205, LoVo, SM-LCN, HCT13, HRT18, HT29-19, CX-1	Variable	LCN2 expression not associated with primary or metastatic cell lines	[44]
	Tumor Tissue	↑	Possible Biomarker, ↑ T-stage, ↓ OS, ↓ DFS	[29,45]
	Subcutaneous Mouse Model	↑	↓ Liver Metastasis	[44]
Endometrial Carcinoma	Cell Lines: HHUA, HEC1A, HEC1B, KLE, RL95-2	↑	↑ Migration, ↑ Chemotherapy Resistance	[46,47]
	Tumor Tissue	↑	Possible Biomarker for Cancer	[46]
Gastric	Tumor Tissue	↑	↓ Invasion, ↑ E-cadherin	[42]
Lung	Cell Line: A549	↑	↑ Radioresistance	[48]
Oral	Cell Line: Ca9-22	↑	↑ Radioresistance	[48]
	Tumor Tissue	↓	Possible Biomarker, ↑ Proliferation, ↑ Migration, ↑ Chemoresistance	[49]
Ovarian	Tumor Tissue	↑	↓ OS, ↓ DFS	[35]
Pancreas	Cell lines Panc-1, MIA PaCa-2, BxP-3, KMP-2, -3, -4, -5, -6, AsPC1, CAPAN1, -2, CFPAC1, COLO347, Hs766T, HPAF, SU.86.86, A818-4, IMIM-PC-2, MDAPanc-3, PaC-3, PaTu-1, PaTu-II, PT45, RWP, SUIT2, T3M4	↑	Tumorigenesis, Metastasis ↑ Invasion, ↑ Adhesion	[27,30,32,37,50,51]
	Tumor Tissue	↑	Poor Prognosis	[27,30,32,50]
	Pancreatic Juice	↑	Possible Biomarker	[52]
	Orthotopic Xenograft	↑	↑Tumor Size, Worse Metastatic Score, ↑ Angiogenesis	[50]
	Plasma		Possible Biomarker	[27,53]
Prostate	Cell Lines: PC3, DU145	↑	↑ Proliferation, ↑ Invasion, ↑ Migration	[54]
	Tumor Tissue	↑	Moderate to Poor Tumor, ↑ Gleason Grade	[54]
Thyroid	Cell Line: FRO	↑	↑ Tumor Colony Formation	[33]
	Tumor Tissue	↑	Possible Biomarker, ↑ Malignant Phenotype	[33,55]
	Orthotopic Xenograft	↑	↑ Tumorigenicity	[33]

Abbreviations: ER: estrogen receptor; PR: progesterone receptor; HER2: human epidermal receptor 2; DSS: disease-specific survival; OS: overall survival; DFS: disease-free survival; SM-LCN: overexpressing LCN2 clones of KM12C.
